# Developing a Novel Mobile App to Support HIV Testing and Pre-Exposure Prophylaxis Uptake Among Men Who Have Sex With Men: Formative and Technical Pilot Study

**DOI:** 10.2196/62830

**Published:** 2025-05-29

**Authors:** Albert Y Liu, Thiago S Torres, Cat-Dancing Alleyne, Janie Vinson, Kelly Bojan, Pedro Alonso Serrano, Temitope Oyedele, Amayvis Garcia, Elizabeth Enriquez-Bruce, Patricia Emmanuel, Jeb Jones, Kathryn E Muessig, Susan P Buchbinder, Patrick Sullivan, Lisa B Hightow-Weidman, Hyman Scott

**Affiliations:** 1 Bridge HIV San Francisco Department of Public Health San Francisco, CA United States; 2 Department of Medicine University of California San Francisco San Francisco, CA United States; 3 Instituto Nacional de Infectologia Evandro Chagas, Fundação Oswaldo Cruz (INI-Fiocruz) Rio de Janeiro Brazil; 4 The Ruth M. Rothstein CORE Center John H. Stroger Jr. Hospital Chicago, IL United States; 5 Center for Dissemination and Implementation Science University of Illinois Chicago, IL United States; 6 Department of Medical Social Sciences Northwestern University Chicago, IL United States; 7 Department of Pediatrics Morsani College of Medicine University of South Florida Tampa, FL United States; 8 Department of Epidemiology Emory University Atlanta, GA United States; 9 College of Nursing Florida State University Tallahassee, FL United States

**Keywords:** HIV testing, sexually transmitted infection testing, pre-exposure prophylaxis, youth, men who have sex with men, sexual minority men, mobile health, HIV prevention, STI

## Abstract

**Background:**

Young sexual minority men (YSMM) are disproportionately impacted by HIV in the United States. HIV or sexually transmitted infection (STI) testing rates and pre-exposure prophylaxis (PrEP) uptake are low in this priority population. Novel strategies are needed to increase access to HIV and STI prevention services among YSMM.

**Objective:**

This study aims to describe the development and assess the feasibility and acceptability of LYNX, a mobile app to increase HIV testing and PrEP uptake among YSMM.

**Methods:**

Informed by the Information-Motivation-Behavioral Skills model, the LYNX app was refined through 4 iterative focus groups in 2 US cities among YSMM aged 15 to 24 years. The LYNX app includes SexPro, an innovative tool that provides a personalized sexual health protection score, a sex diary to track sexual partners, HIV and STI testing information and reminders, access to home HIV and STI test kits, and geospatial-based testing and PrEP clinic site information. The refined app was then tested for feasibility and acceptability in a 2-month technical pilot. Baseline and 2-month follow-up assessments and exit interviews were completed. Self-reported app acceptability and use based on paradata were reported.

**Results:**

In iterative focus groups among 30 participants (age: mean 20, SD 3 years; Black: 12/30, 40%; Hispanic or Latinx: 13/30, 43%), the app’s design was well-received. Participants recommended providing information on how the SexPro score was calculated and how they could improve their score, changes to the language in the sex diary tailored for YSMM, providing a chat feature to facilitate communication between staff and app users, and gamification features to increase overall youth engagement with the app. These recommendations were incorporated into the app. In the technical pilot among 17 participants (age: mean 22.4, SD 1.6 years; Black: 4/17, 24%; Hispanic or Latinx: 8/17, 47%), the mean system usability score was 70 out of 100, falling in the “good” range. Use of the app was high over the 2-month pilot (app opened an average of 8.5, SD 8.0 times with an average duration of 3.8, SD 3.2 min/session), indicating good feasibility. The most commonly used features included the testing feature (n=15, 100%), activity calendar (n=14, 93%), and diary (n=13, 86%). Overall, 11 (79%) participants were likely to continue using LYNX, and 10 (71%) participants were likely to recommend it to a friend. In exit interviews, there was a high level of acceptability of the content, interface, and features of the LYNX app.

**Conclusions:**

Following a user-centered design approach, we tailored the LYNX app to increase HIV and STI testing and PrEP uptake among YSMM in the United States. Our positive findings support further testing of this mobile health tool in an upcoming effectiveness trial in broader youth populations.

**Trial Registration:**

ClinicalTrials.gov NCT03177512; https://clinicaltrials.gov/study/NCT03177512

**International Registered Report Identifier (IRRID):**

RR2-10.2196/10659

## Introduction

### Background

Young sexual minority men (YSMM) are disproportionately impacted by HIV in the United States. In 2021, 19% of the estimated 32,100 new HIV cases in the United States were among young people aged 13-24 years, with YSMM accounting for 82% of new HIV diagnoses in this age group [1]. YSMM of color are particularly impacted by HIV, with Black and Latino sexual minority men (SMM) accounting for 53% and 28% of infections among YSMM, respectively, in 2021 [1]. There is an urgent need to expand access to effective HIV prevention strategies in this priority population.

### HIV Testing and Pre-Exposure Prophylaxis Use Among YSMM

HIV testing is the gateway to the HIV treatment and prevention continua, and it affords an important opportunity to offer timely treatment for YSMM living with HIV and for linkage to effective preventive strategies for those who test HIV negative. Although the Centers for Disease Control and Prevention recommends at least yearly HIV testing for SMM, in a recent national web-based survey, only 40% of YSMM reported testing in the past year, and 45% had never tested in their lifetime [2]. Young people are the least likely to be aware of their HIV status, with only 56% of US youth living with HIV aware of their diagnosis in 2022 [3]. Despite YSMM having among the highest annual sexually transmitted infection (STI) rate among any age group [4], STI testing rates are also low [5], with only a third of YSMM reporting STI testing in the last year [6]. Low perceived risk, lack of symptoms, limited knowledge of STIs, and lower access to health care providers have been identified as barriers to HIV and STI testing [7-9].

Numerous studies have demonstrated the effectiveness of pre-exposure prophylaxis (PrEP) for HIV prevention [10-12], now one of the pillars of the Ending the HIV Epidemic Initiative in the United States; however, uptake of PrEP has been low among YSMM. According to national prescription data, youth aged 15 to 24 years had the greatest unmet need for PrEP among all age groups, with a PrEP-to-need ratio of 9, indicating that for every person in this age group diagnosed with HIV, there were only 9 people using PrEP, compared to a PrEP-to-need ratio of 17 for people aged 35 to 44 years [13]. Demonstration projects also highlight challenges with PrEP uptake. In the Adolescent Medicine Trials Network for HIV Interventions (ATN), 110 study of YSMM aged 18 to 22 years, PrEP uptake was only 16%, and PrEP adherence was lower among Black YSMM and declined overall during follow-up, particularly with less frequent visits [14]. A number of factors contribute to low PrEP uptake, including low awareness and knowledge about PrEP, limited access to PrEP providers and clinics, deficits in self-perceived risk, HIV- and PrEP-related stigma, and medical mistrust, especially among YSMM of color, and highlight the importance of innovative strategies to increase testing and PrEP uptake tailored for YSMM [15-17].

### Potential of Mobile Health Technologies

Mobile technologies have enormous potential to reach and engage YSMM in HIV prevention [18-22]. Mobile phone use is nearly ubiquitous among youth, and youth are more likely to use their mobile devices for more activities, such as downloading mobile apps, internet access, social media, and accessing health information, compared to people in older age groups [23,24]. The expansion of smartphones has increased the possibilities of dynamic, mobile phone–based HIV prevention interventions. Particularly, a mobile phone app designed to increase HIV testing and PrEP uptake could be highly scalable and has great potential to expand access to and uptake of prevention services among youth.

**Figure 1:**
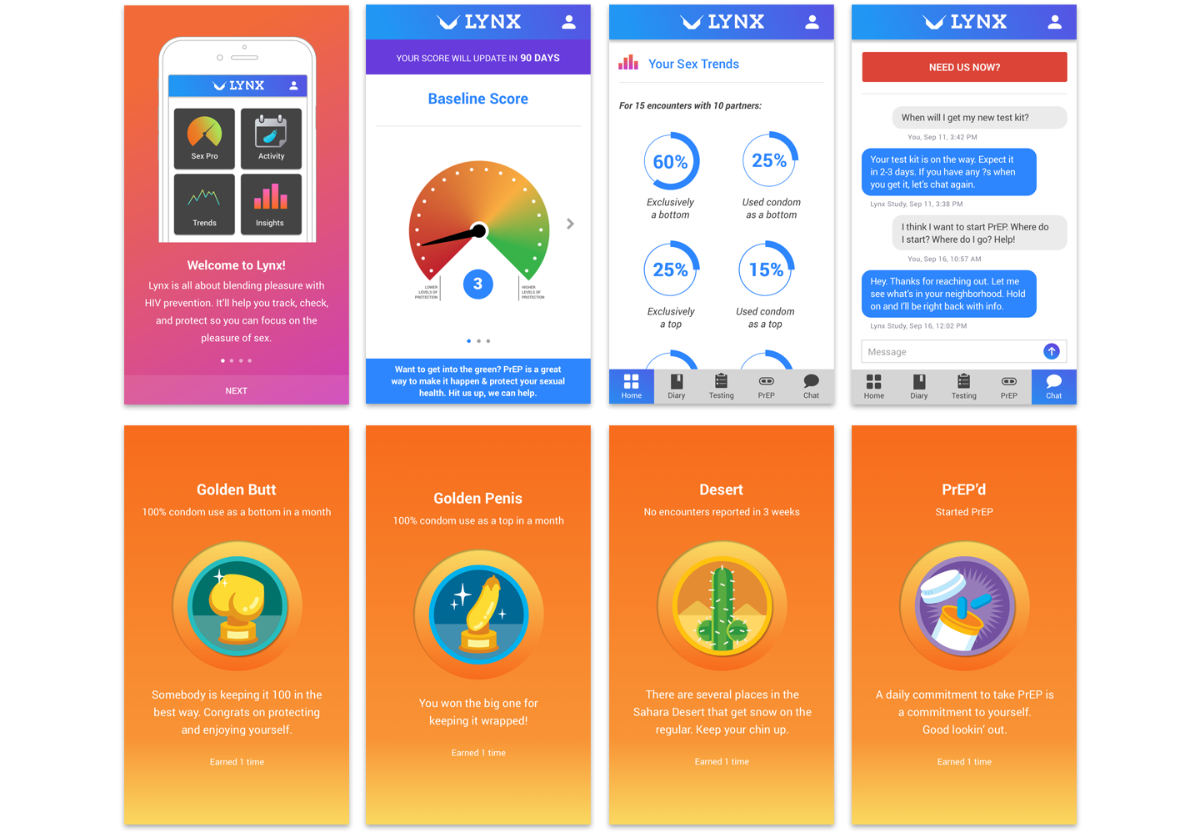
LYNX app screenshots. PrEP: pre-exposure prophylaxis.

Using the Information-Motivation-Behavioral Skills (IMB) model [25], we previously developed the LYNX app, a highly interactive mobile app to promote accurate risk perception and increase HIV and STI testing and linkage to care, which has been previously described [26]. Components of the app are shown in Figure 1 and include (1) SexPro (Sexual Health Promotion), an innovative tool that provides a personalized HIV risk score displayed on a “speedometer” (1-20), with a higher score representing higher levels of protection [27]; (2) a sex diary to help users track their sexual partners and encounters, which are then displayed in a calendar; (3) HIV and STI testing information and reminders; and (4) ability to order home HIV point-of-care self-test kits (Oraquick) and home STI testing collection kits (self-collected specimens mailed to a central laboratory for testing) through the app and access the geospatial-based testing site and linkage to HIV care information. To protect participant privacy, the app is accessed through a secure password-protected log-in, and information collected through the app is encrypted and stored in a secure environment that supports Health Insurance Portability and Accountability Act (HIPAA) compliance.

In this study, we describe the results of a 2-phase study to develop and refine the LYNX app tailored for YSMM through iterative focus groups and an open technical pilot. This study was conducted within the National Institutes of Health’s ATN as part of the University of North Carolina and Emory Center for innovative technology (iTech), which had the overall goal to develop technology-focused interventions addressing the HIV prevention and care continuum for youth [28].

## Methods

### Study Population

Eligible participants included cisgender men who were aged 15 to 24 years, self-reported being HIV negative or HIV status unknown at screening (technical pilot only), not currently taking PrEP (technical pilot, as participants are testing an app, which assists with PrEP uptake), fluent in English, owned an iOS or Android mobile phone, and had self-reported evidence of being at risk for HIV acquisition (detailed risk criteria are described in the study by Liu et al [26]). Focus group participants were able to participate in the technical pilot if they remained eligible.

Through the National Institutes of Health’s ATN iTech U19, participants were recruited across 2 sites—Chicago, Illinois (study site: CORE Center), and Tampa, Florida (study site: University of South Florida). Participants for focus groups and the technical pilot were recruited through a variety of strategies, including web-based and social media postings (eg, Craigslist, Grindr, and Facebook advertisements); distributing posters, flyers, and palm cards about the study; direct outreach at local venues frequented by YSMM (eg, community-based organizations, schools, social clubs, health fairs, and balls); clinic-based recruitment; and participants in prior studies. To ensure inclusion of youth most impacted by HIV, we oversampled YSMM of color, with a goal of enrolling two-thirds of the cohort as YSMM of color and at least one-quarter as Black YSMM. Oversampling was accomplished by focusing recruitment efforts on venues more likely to be frequented by YSMM of color.

### Iterative Focus Groups for App Refinement

After an initial prototype of the LYNX app was developed by the study team, we conducted 2 rounds of formative work through a series of 4 focus groups and individual interviews with 30 YSMM across our 2 iTech sites, which informed iterative development of the LYNX app. YSMM participated in 1 of the 4 groups, which each met 2 times. Participants unable to be scheduled for a focus group were offered an individual interview, with the same content covered in both focus groups and interviews. Groups were divided by age strata (15-18 years, 19-24 years, etc), and focus groups were conducted in a private room at each site by a research staff member trained in qualitative methods and group facilitation. All participants completed a written consent or assent process before initiation of the focus group. During the focus group discussion, participants were shown how to download and install the app and were given a demonstration of key features of the LYNX app. The home HIV and STI testing process was also explained, and participants were shown a test kit. New app components were developed to increase PrEP uptake and included a PrEP Facts page with information about PrEP, a map showing local PrEP clinics in an area, PrEP video testimonials of youth sharing their experiences about taking PrEP, and a feature of SexPro showing how PrEP can improve one’s score. Participants were asked to give feedback on each component of the app and suggestions for improvement. At the end of the focus group, participants completed a brief web-based survey to assess demographic characteristics and use of technology to help contextualize the focus group discussion.

Members of the iTech Analytic Core reviewed transcripts and identified emergent themes. We conducted thematic analysis using a deductive approach to code data for app acceptability overall and for each component, with a focus on functionality, appearance, usability, and privacy and confidentiality. Key findings are described in the text using representative quotes and were used to refine the LYNX app before initiation of the technical pilot.

### Changes to the LYNX App

On the basis of feedback from the focus groups and interviews, we made a list of recommended changes to the app. The study team then prioritized changes based on the perceived impact of the changes on the usability and acceptability of LYNX, the likelihood of increasing HIV and STI testing and PrEP uptake, and the technical feasibility of the modifications. We then worked with our app developer to implement these revisions to the LYNX app.

### Technical Pilot to Assess Feasibility and Acceptability of the LYNX App

After refining the LYNX app based on our formative work, we conducted a 2-month technical pilot among 17 participants to assess the feasibility and acceptability of the LYNX app and to elicit additional feedback for app improvement. Eligible participants who enrolled in the study completed a web-based survey on sociodemographics, risk behaviors, and use of technology. Study staff assisted participants in downloading the LYNX app and were shown how to use key features of the app using an onboarding guide. Participants were encouraged to use all components of the app over the next 2 months, including ordering and using the HIV and STI test kit.

Upon completion of the technical pilot at month 2, participants were asked to complete a brief web-based survey to assess preliminary usability and acceptability of the app using the System Usability Scale (SUS), a validated tool assessing various domains of the app with demonstrated high internal consistency across several studies [29]. The SUS is a 10-item scale that measures a system’s usability on a scale of 0 to 100. Feasibility was assessed using app analytics to determine whether the app was used, how many times it was used, and which components were used most and least frequently. Feasibility was a priori defined as having at least 60% of individuals open the LYNX app at least once after introduction to the app by research staff.

Each participant also completed an exit interview to provide feedback on functionality, technical performance, errors and bugs encountered, overall experiences using the app, feasibility and acceptability of methods to confirm HIV testing results, and feedback for further refinement. Exit interviews were conducted remotely with qualitative-trained study staff from the iTech Analytic Core using a HIPAA-compliant videoconferencing technology. Debriefing reports capturing the quality and key findings were completed after each interview. Interviews were also transcribed and analyzed using the same approach outlined earlier.

### Ethical Considerations

The study procedures were reviewed and approved by the University of North Carolina Institutional Review Board (IRB) as a single IRB-of-Record (approval number: 17-0170). IRB authorization agreements with all participating research entities were enacted. Written informed consent was obtained before conducting any study activities. All study data were identified by a coded number only to maintain participant confidentiality. The LYNX protocol is registered at ClinicalTrials.gov (NCT03177512). For iterative focus groups, participants received US $50-$60 for completing each focus group or interview, depending on local site standards. For the technical pilot, participants received US $50-$60 for completing the baseline visit, US $25-$30 for completing the 2-month survey, and US $50-$60 for completing the final exit interview. Additionally, at one site, a $30 gas card for round trip transportation was provided. Incentive amounts varied by site and were based on local site standards.

## Results

### Iterative Focus Groups for App Refinement

Characteristics of the 30 focus group participants, 18 (60%) in Chicago and 12 (40%) in Tampa, are shown in Table 1. Mean age was 20 (SD 3.0) years; 13 (43%) self-identified as Hispanic or Latinx, 18 (60%) completed high school or lower, and 13 (43%) were currently in school. A substantial proportion reported having health insurance (n=21, 70%), ever tested for HIV (n=26, 87%), tested for other STIs in the past year (n=22, 73%), and ever taken PrEP (n=16, 53%). Most participants reported using iOS (n=20, 67%), receiving health information through a website or an app on a mobile phone (n=23, 77%), and that they thought receiving health information through a mobile app would be of interest to YSMM (n=27, 90%).

**Table 1:** Characteristics of focus group participants for the development of an HIV prevention mobile app from Chicago, Illinois, and Tampa, Florida (N=30).

Characteristics		Values
**City, n (%)**		
	Chicago	18 (60)
	Tampa	12 (40)
Age (y), mean (SD)		20 (3.0)
**Sexual orientation, n (%)**		
	Gay	21 (70)
	Bisexual	9 (30)
**Race** **, n (%)**		
	Asian	3 (10)
	Black	12 (40)
	Hispanic or Latinx	13 (43)
	Mixed race	4 (13)
	White	6 (20)
	No answer	1 (3)
**Schooling, n (%)**		
	Lower than high school	7 (23)
	Completed high school	11 (37)
	Some college	8 (27)
	College completed	4 (13)
	Currently in school	13 (43)
Health insurance, n (%)		21 (70)
Insurance coverage through parent or guardian, n (%)		10 (33)
Primary partner, n (%)		10 (33)
Number of male partners (past 6 months), mean (SD)		5 (6.0)
Number of male sexual partners met through internet (in the past 6 months), mean (SD)		5 (8.0)
**HIV testing, n (%)**		
	Never	4 (13)
	Past 3 months	18 (60)
	>3 months	8 (27)
Tested for other STIs^a^ (in the past year), n (%)		22 (73)
PrEP^b^ awareness before the study, n (%)		29 (97)
**Ever taken PrEP, n (%)**		
	Never	14 (47)
	Yes, but not currently taking PrEP	5 (17)
	Yes, I am currently taking PrEP	11 (37)
How likely to use PrEP in the future (extremely likely or likely)^c^, n (%)		18 (60)
Previously been in a PrEP research study, n (%)		21 (70)
**Operating system, n (%)**		
	iOS	20 (67)
	Android	10 (33)
Share phone with someone, n (%)		2 (7)
Phone disconnected (past 12 months), n (%)		14 (47)
Phone lost or stolen (past 12 months), n (%)		9 (30)
**Ever used internet for, n (%)**		
	Tracking health behaviors	20 (67)
	Acquiring information about HIV or other STIs	13 (43)
	Acquiring other health or medical information	15 (50)
	Sending reminders	17 (57)
**Most common ways for getting health information** **, n (%)**		
	Website or app on mobile phone	23 (77)
	Website or app on laptop or desktop computer	15 (50)
	Health care providers	21 (70)
Think receiving health information through a mobile app would be of interest to young MSM^d^, n (%)		27 (90)

^a^STI: sexually transmitted infection.

^b^PrEP: pre-exposure prophylaxis.

^c^Among all participants.

^d^MSM: men who have sex with men.

Overall, the app was well-received by participants, and the design was thought to be aesthetically pleasing and intuitive. Participants saw the app’s utility and thought that it would be a helpful tool for people to engage in their sexual health. A participant in Tampa stated the following:

I see this as a fitness health app, but for sexual health. So that does seem useful.

Participants suggested that the goal of the app be made clear as part of the onboarding process, which was added after the first round of focus groups.

Regarding SexPro, participants felt the numeric score and the color coding (red for low score indicating low protection from HIV, yellow or orange for intermediate score indicating moderate protection, and green for a high score indicating high protection) were important to provide a more robust understanding of what their score meant. One participant in Chicago described it as follows:

The higher the number the better, even 12 versus 9 just sounds and feels better. Like if I have a 12, I feel like I’m getting there, I can start to get better. If I see a 7, I just think “fuck!” I’m screwed. Especially versus if you just showed me that scale with no numbers, I would be worried, I would think I’m not protected. I would think crap, I’m screwed.

Another participant in Chicago stated the following:

Yeah, if it’s orange I wanna do something to get it to go up (to green). But if it’s red you might think you are going to have to make drastic lifestyle changes to get out of the red zone.

Participants thought that seeing the score could help motivate a change in behavior, as a participant from Tampa stated the following:

Seeing the score would be good to motivate you to maybe change your life, or your habits, so you can increase your score.

Several participants suggested adding a summary to help users understand what went into their score, as well as suggestions for how to improve their score. A participant in Tampa said the following:

I think it would be useful to see, rather than a number, but like why I got a number. If I compared my score to a friend’s, and they had a different number—we could compare, like, this is what I did, this is what you did, that’s why a summary page would be more useful.

Participants also liked the sexual diary component of the app. A participant in Chicago said the following:

I do think that’s kind of useful, so you can keep track of what you’re doing, or you know, if you need to “slow your ho down”—I feel like you can be able to hide it, so people don’t see it, or call your 5-star person up.

Another participant in Chicago commented the following:

The diary makes it fun, and not just a health app. I would look forward to looking back at it because of all the fun comments I made in it. It makes being safe fun to me.

One participant liked having a separate place to track their sexual partners without having the information living in their phone’s contact list, saying the following:

Let’s just say I was raped, or I had lost my phone or whatever, I could download the app on my new phone where I had backed it up and I would still have that person’s information in the app, because I don’t usually add people in my phone contact list. It’s good information to have and keeps it all private.

Participants also liked the notes section of the diary, as it made the diary seem less like a tool of objectification, with a participant in Tampa saying the following:

People judge people anyway, but having like the notes makes it more personal... you can go back and look at the things you said to remember, or things about that person you want to recall later, even if it’s like, you know, their apartment was dirty.

Participants did recommend changing the terminology for describing partner types in the diary. For example, they found “no strings attached” to be confusing and suggested changing this to “one night stand.”

Most participants felt comfortable using the home HIV and STI self-collection kits and saw value in this service, although YSMM aged 15 to 18 years expressed some concerns regarding potential errors with using the STI test kits and felt the shipping process was cumbersome and may be a deterrent for youth. Participants remarked that testing services were limited in their area (particularly Tampa). They reported that the few places that offered services had limited hours that conflicted with work and school schedules. A participant in Chicago said the following:

This option is a lot better especially if you are scared to go to a doctor. That’s reassuring.

They highlighted the importance of confidentiality. A participant in Tampa said the following:

As long as the packaging is confidential that came to my home. I’m totally fine to do a care kit like that. Especially since it’s free to request and ship.

However, some participants felt that testing at home would mean they would miss out on the opportunity to build rapport with their medical provider. In addition, some participants reported concerns with collecting the specimens and preparing the kits for shipment, as a participant in Chicago said the following:

For me it’s easier to go to the doctor’s office, then I don’t have to worry about messing something up or anything like that.

If someone were to receive a positive HIV test result, participants wanted immediate access to a provider who could offer supportive counseling. They also suggested adding a chat function to communicate with the clinic.

Participants appreciated the PrEP components of LYNX, including the ability to find local PrEP clinics in their area; however, they recommended that the goal of increasing PrEP uptake be made clearer in the onboarding section of the app. One focus group participant in Tampa said the following:

As far as like the HIV and like the prep, thing when I first downloaded it, I didn’t really see it as that being like the focus. I thought like the focus was more of tracking your partners.

While some participants liked showing how one’s SexPro score increases on PrEP, they recommended including information about other non-PrEP–related factors that could influence their SexPro score, as described by another participant in Tampa:

Yeah like if swiping right included other things like let’s see what your score would look like if you used like more condoms, or you know decreased your number of partners as well. Like different factors not always just, well if you were on PrEP this is what would like... obviously PrEP is not the only thing that’s going to bring up your level and increase your protection.

Participants were also interested in gamification and receiving feedback from the app based on the data they entered into the app. When shown examples of badges in the focus groups, one participant said the following:

It’s a pat on the back. It’s cute! You are being rewarded for being a ho! (followed by laughter)

They liked the golden penis badge and suggested having a “gold butt” badge as a complement (Figure 1). They also recommended creating badges to keep track of PrEP pill taking and having a “desert badge” when they are not having sex. They also liked the idea of having a trends and insights page, which would provide additional feedback on their trends in sexual behaviors.

### Changes to the LYNX App

On the basis of the feedback from the focus groups, we made several revisions and additions to the LYNX app, guided by the IMB model. Regarding “information,” several welcome screens were added to the onboarding process to clarify the goals of the app, including the message “to marry pleasure with prevention,” which was a theme that emerged from the focus groups, as well as clarifying the goal of increasing PrEP uptake. To provide more information about PrEP, a PrEP Facts page was added that included answers to frequently asked questions about PrEP. Regarding SexPro, we added information on behaviors contributing to the calculation of their SexPro score, with an option to click on “Learn why these matter,” which provides a more detailed description of how different behaviors change one’s risk for HIV acquisition. We also added information about how participants could improve their score and how their score would be impacted by taking PrEP. To provide additional feedback to participants on the data they entered into LYNX, we also created a trends page that summarized different sexual behaviors and encounters (eg, you were exclusively a bottom 50% of the time) and an insights page that summarized characteristics of their sexual partners (gender identity, partner type, partner HIV status, and number of repeat partners). Regarding “motivation,” we added video testimonials of YSMM on their experience taking PrEP to help motivate YSMM to consider taking PrEP. We also added several gamification features to the app, including the ability to earn different types of badges (Figure 1), and a “top 5” feature for the 5 most highly rated partners and 5 most highly rated sexual encounters. In addition, the language in the sex diary was modified based on input from the focus groups (eg, changing “no strings attached” to “one night stand”) and the notes section was included in the final design to allow participants to enter free text to capture anything they would like to remember about the partner. Regarding Behavioral skills, we added the PrEP locator function to allow participants to find a local PrEP clinic that served youth. We also added a chat function to allow participants and study staff to send and receive in-app messages for additional guidance and support, as well as a “need us now” button in red at the top of the chat screen, which would allow participants to call an on-call provider available at all times. This chat function was real time and monitored by study staff (including study clinicians), who responded to participants within 24 business hours. Finally, we engaged a copywriter who simplified the language, using fewer words, and made the language in the app more appealing for youth. There were a few suggestions that were not incorporated into the app due to feasibility considerations but noted for future iterations of the app, including log-in via touch ID or social media log-in (instead of passcode), adding a group sex option in the sex diary, and adding dosing reminders for people who started taking PrEP.

### Technical Pilot to Assess Feasibility and Acceptability of the LYNX App

From April to June 2018, a total of 17 participants were enrolled in the pilot study: 8 (47%) in Chicago and 9 (53%) in Tampa. Table 2 describes baseline characteristics of participants included in the technical pilot study. Overall, the mean age was 22.4 (SD 1.6) years; 8 (47%) self-identified as Hispanic or Latinx, and 5 (29%) completed high school or lower. The majority were currently employed (15/17, 88%), reported low income (12/17, 71%), lived with another person (14/17, 82%), currently had health insurance (10/17, 59%), and did not have a primary health care provider (12/17, 71%). The mean number of partners was 3.1 (SD 2.5) in the past 3 months, and 11 (65%) participants reported condomless sex. A total of 5 (29%) participants never tested for HIV, and only 1 (6%) tested in the past 3 months. For other STIs, 9 (53%) participants had never tested before and 8 (47%) tested >3 months ago. Regarding prevention technologies, 11 (65%) and 15 (88%) heard about HIV self-testing and PrEP before the study, respectively. Most participants (n=10, 59%) reported using the iOS mobile phone operating system, had a phone contract billed monthly (n=9, 53%), and used apps more than once per week (n=14, 82%).

A total of 15 (N=17, 88%) participants completed the follow-up visit. The mean SUS score was 70.4 (SD 17.2), corresponding to a rating in the good range (Table 3) [30]. The highest score was on item “I thought the system was easy to use” (mean 4.3, SD 0.9), and the lowest score was on item “I needed to learn a lot of things before I could get going with LYNX” (mean 2.2, SD 1.4). Most reported using the LYNX app at least once a week (8/14, 57%); that they would be somewhat likely, likely, or very likely to continue using the app (11/14, 79%); and would definitely recommend it to a friend in need of similar help with getting tested for HIV and STIs and accessing PrEP (10/14, 71%). A total of 43% (6/14) of participants ordered an HIV test kit, and 36% (5/14) ordered an STI test kit.

**Figure 2:**
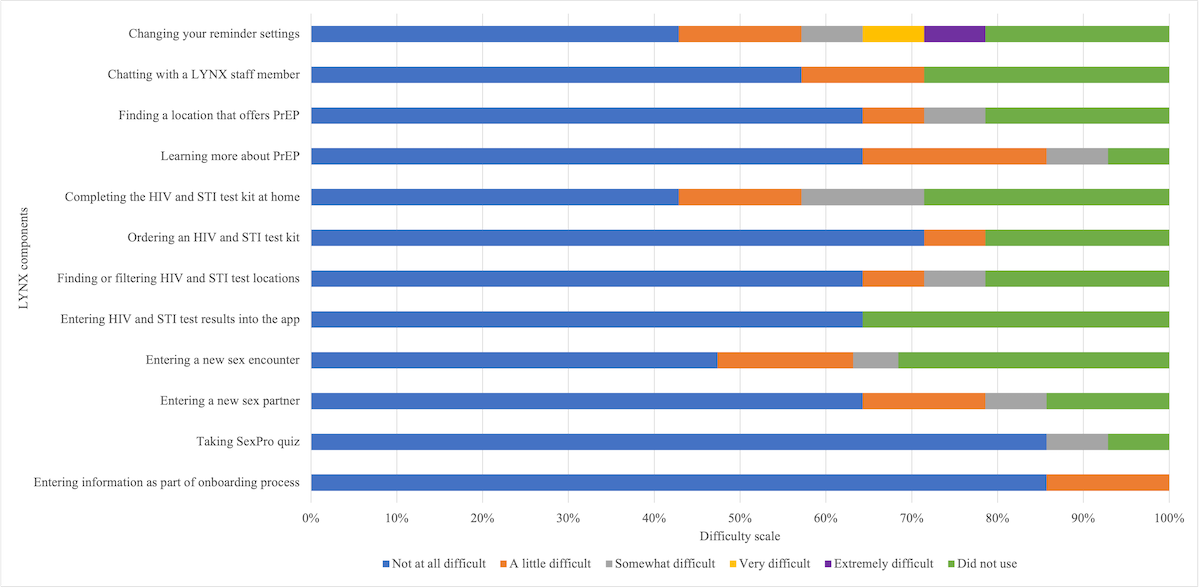
Ratings of ease of use of the components of the LYNX app among technical pilot participants in Tampa and Chicago. PrEP: pre-exposure prophylaxis; STI: sexually transmitted infection.

**Figure 3:**
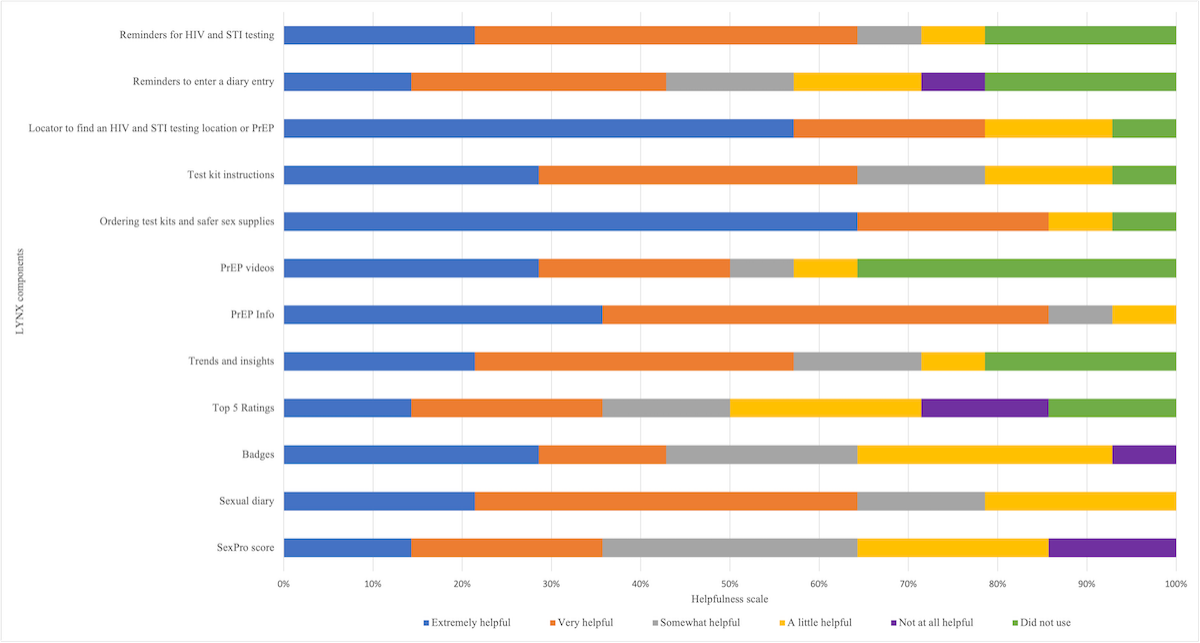
Ratings of helpfulness of the components of the LYNX app among technical pilot participants in Tampa and Chicago. PrEP: pre-exposure prophylaxis; STI: sexually transmitted infection.

**Figure 4:**
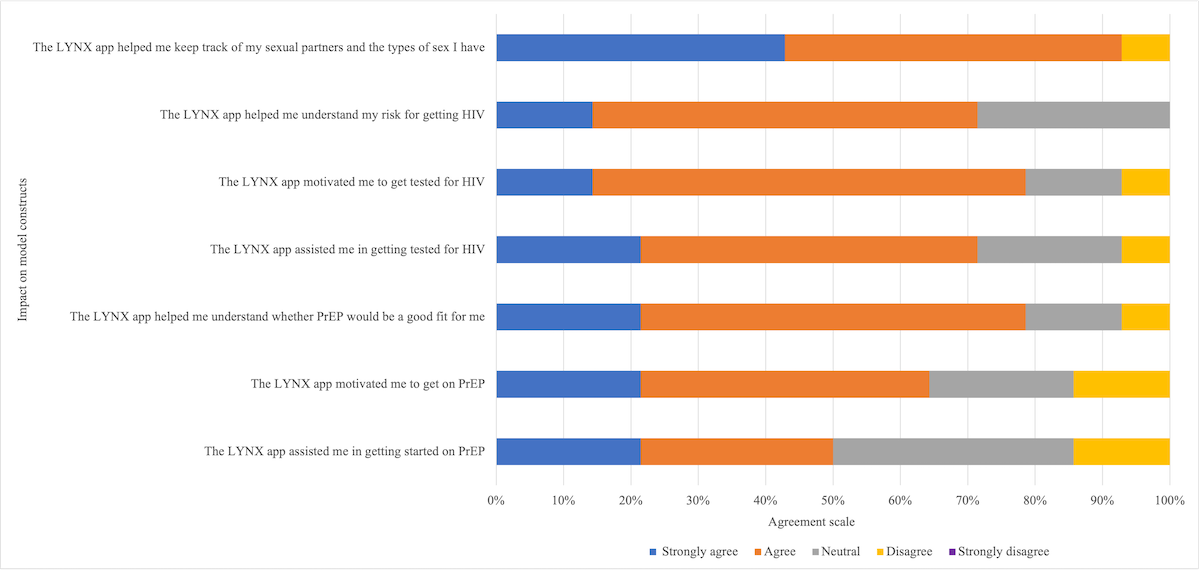
Impact of the LYNX app on Information-Motivation-Behavioral Skills model constructs among technical pilot participants in Tampa and Chicago. Responses ranged from strongly agree to strongly disagree. No participants indicated “strongly disagree” to any of the response items. PrEP: pre-exposure prophylaxis.

Most participants rated all components of LYNX as not at all difficult or a little difficult to use (Figure 2). Almost 86% (12/14) of participants reported the components “Taking SexPro Quiz” and “Entering information as part of the onboarding process” as not at all difficult. More than half of the participants considered all components at least somewhat helpful (Figure 3). “Ordering test kits and safer sex supplies” was considered extremely helpful for 64% (9/14) of respondents. More than half of the participants strongly agreed or agreed with statements related to IMB model impact (Figure 4). More than 90% (13/14) strongly agreed or agreed that “the LYNX app helped me keep track of my sexual partners and the types of sex I have” (13/14, 93%). More than 70% (10/14) strongly agreed or agreed that “The LYNX app helped me understand my risk for getting HIV” (10/14, 72%) and “The LYNX app helped me understand whether PrEP would be a good fit for me” (11/14, 79%) and half strongly agreed or agreed that the app helped them in starting PrEP (7/14, 50%).

Participants opened the app, on average, 8.5 (SD 8.0, range 1-31) times during the 2-month pilot intervention period, with an average duration of 3.8 (SD 3.2, range 0.2-11.8) minutes per session. Across all participants, the cumulative time spent in the app ranged from 0.6 to 183.7 minutes, with a median of 16.5 (IQR 5.45-40.8) minutes. The mean time between enrollment and first app log-in was 5.7 (SD 17.2, range 0-67) days. The mean number of features accessed was 9.5 (SD 4.8, range 0-14) out of 16 features. The “testing” feature was used by all participants (15/100, 15%), followed by “Activity Calendar” (14/15, 93%) and “Diary” (13/15, 87%; Table 4). The most visited features were “Home Screen” (240 visits), “Diary” (189 visits), and “Testing” (174 visits).

**Table 2:** Baseline characteristics of technical pilot participants for an HIV prevention mobile app from Chicago, Illinois, and Tampa, Florida (N=17).

Characteristics		Values
**City, n (%)**		
	Chicago	8 (47)
	Tampa	9 (53)
Age (y), mean (SD)		22.4 (2)
**Sexual orientation, n (%)**		
	Gay	11 (65)
	Bisexual	5 (29)
	Queer	1 (6)
**Race, n (%)**		
	Asian	1 (6)
	Black	4 (24)
	Hispanic or Latinx	8 (47)
	White	10 (63)
**Schooling, n (%)**		
	Completed high school or lower	5 (29)
	Some college	7 (41)
	Completed college	5 (29)
	Currently in school	7 (41)
Current employed, n (%)		15 (88)
**Income (self-defined), n (%)**		
	Low	12 (71)
	Middle	4 (24)
	Declined to answer	1 (6)
**Relationship status, n (%)**		
	Single	4 (24)
	Casually dating	5 (29)
	Boyfriend, girlfriend, partner, or lover	8 (47)
**People living with, n (%)**		
	Alone	3 (18)
	1-2	5 (29)
	3-5	7 (41)
	>5	2 (12)
**Health insurance, n (%)**		
	No	7 (41)
	I have my own	6 (35)
	I am covered by my parent or guardian	4 (24)
Primary health care provider, n (%)		5 (29)
Number of male partners (past 3 months), mean (SD)		3.1 (3)
Condomless sex (past 3 months), n (%)		11 (65)
**HIV testing, n (%)**		
	Never	5 (29)
	Past 3 months	1 (6)
	>3 months	11 (65)
**Ever tested for other STIs^a^, n (%)**		
	Never	9 (53)
	Past 3 months	0 (0)
	>3 months	8 (47)
**HIV self-testing, n (%)**		
	Never heard about it	6 (35)
	Heard before, but never used it	9 (53)
	Used previously	2 (12)
PrEP^b^ awareness before the study, n (%)		15 (88)
**Interested in using PrEP, n (%)**		
	Not at all interested	2 (12)
	A little interested	5 (29)
	Somewhat interested	4 (24)
	Very interested	4 (24)
	Extremely interested	1 (6)
**Operating system, n (%)**		
	iOS	10 (59)
	Android	7 (41)
Share phone with someone, n (%)		3 (18)
**Service, n (%)**		
	Monthly	9 (53)
	Prepaid	2 (12)
	Shared plan	6 (35)
Phone disconnected (past 12 months), n (%)		3 (18)
**How often use apps, n (%)**		
	≤1 per week	3 (18)
	>1 per week	14 (82)

^a^STI: sexually transmitted infection.

^b^PrEP: pre-exposure prophylaxis.

**Table 3:** System Usability Scale (SUS) and app use among technical pilot participants in Tampa and Chicago (N=14).

SUS^a^		Values
**Items, mean (SD)**		
	1: I think that I would like to use LYNX on a regular basis.	3.5 (1.3)
	2: I found LYNX unnecessarily complex.	2.4 (1.3)
	3: I thought the system was easy to use.	4.3 (0.9)
	4: I think that I would need the support of a technical person to be able to use LYNX.	2.3 (1.4)
	5: I found the various functions in LYNX were well integrated.	4.0 (1.4)
	6: I thought there was too much inconsistency between different parts of LYNX.	2.6 (1.2)
	7: I would imagine that most people would learn to use LYNX very quickly.	4.2 (1.1)
	8: I found LYNX very cumbersome to use.	2.5 (1.2)
	9: I felt very confident using LYNX.	3.9 (1.1)
	10: I needed to learn a lot of things before I could get going with LYNX.	2.2 (1.4)
SUS total score, standardized (0-100), mean (SD)		70.4 (17.2)
**In the past 2 months, on average, how often did you use the LYNX app?, n (%)**		
	Once a month or less	1 (7)
	A few times a month	5 (36)
	Once a week	5 (36)
	A few times a week	2 (14)
	Once a day	1 (7)
**How likely would you be to continue using the LYNX app if it were available?, n (%)**		
	Very unlikely	2 (14)
	Unlikely	1 (7)
	Somewhat unlikely	0 (0)
	Neutral	0 (0)
	Somewhat likely	4 (29)
	Likely	4 (29)
	Very likely	3 (21)
**If a friend were in need of similar help with getting tested for HIV or STIs^b^ and accessing PrEP^c^, would you recommend the LYNX app to him?, n (%)**		
	No, definitely not	0 (0)
	No, I do not think so	0 (0)
	Yes, I think so	4 (29)
	Yes, definitely	10 (71)

^a^Scores range from 1 (strongly disagree) to 5 (strongly agree).

^b^STI: sexually transmitted infection.

^c^PrEP: pre-exposure prophylaxis.

**Table 4:** Frequency of features being viewed or accessed by participants in a pilot evaluation of a mobile HIV prevention app from Tampa and Chicago (N=15).

Feature	Unique users, n (%)	Visits, n (%)
Testing page for logging HIV and STI^a^ testing and ordering a test kit	15 (100)	175 (17)
Activity Calendar showing partner encounters	14 (93)	41 (4)
Diary to view and add new partners and encounters	13 (86)	189 (18)
Insights on partner types and HIV status	12 (80)	25 (2)
Navigation Chat: click to page to chat with LYNX team	12 (80)	72 (7)
Navigation PrEP^b^: click to PrEP information page	12 (80)	107 (10)
Sex trends, including sexual activities and condom use	11 (73)	31 (3)
Earned badges based on app use	10 (66)	23 (2)
Profile, including account details and setting reminders	10 (66)	19 (1)
SexPro page to view the current score and explanation	9 (60)	27 (2)
Top 5 partners and encounters	9 (60)	19 (1)
Encounter: recording a new entry in sex diary	6 (40)	24 (2)
PrEP Facts, including frequently asked questions about PrEP	4 (26)	15 (1)
PrEP Videos: testimonials from YSMM^c^ on their PrEP experience	4 (26)	12 (1)
Testing and PrEP Map to search for testing and PrEP clinics	0 (0)	0 (0)

^a^STI: sexually transmitted infection.

^b^PrEP: pre-exposure prophylaxis.

^c^YSMM: young sexual minority men.

Among the 15 participants enrolled in the technical pilot, 9 (60%) participants completed an HIV test during the pilot, including 4 (27%) who tested at home. In addition, 4 (27%) participants started PrEP during the pilot.

Among 17 pilot study participants, 14 (82%) completed follow-up exit interviews. Overall, there was a high level of acceptability of content, interface, and features of the LYNX app, although some participants reported that they did not use all the app features (eg, sex diary) due to being in a monogamous relationship. One participant in Tampa noted the following:

I think that this app is so beneficial for like young gay men...they are scared and not confident in talking about this stuff and they don’t have anyone to talk to...I think that’s one thing that’s really like important for this app because it has the answers, it has where to go, it has everything you need to know when you don’t have the answers.

Most participants liked the SexPro feature and thought that it accurately reflected their risk; however, most did not look back at the score during the pilot and did not believe that the SexPro score changed their level of interest in PrEP. Most participants liked the diary, trends, and insights features, as they helped them keep track of their partners and encounters and reflect on their sexual behaviors. One participant in Tampa said the following:

Yeah. I actually really thought the app is great. I lost tracking, my—like just my encounters and thought that was really beneficial especially like going back in time to kind of look at it because I don’t know, it just keeps your eyes open about what you’re doing.

However, one participant felt that the diary was judgmental and objectifying of partners and was concerned that it would hurt a partner’s feelings if they somehow saw it. Participants felt the diary was more helpful in tracking one-time encounters with casual partners, and this feature was less helpful for those in monogamous relationships. One participant suggested being able to look at data from a specific time frame (rather than cumulative partners and encounters), and several participants wanted the ability to edit previously entered entries. While most participants did not use the chat function, they liked that the feature was available, especially the “need us now” button. Many participants did not recall receiving notifications and reminders for HIV and STI testing, and would have preferred either weekly or monthly reminders. A few participants reported technical issues, such as receiving notifications at unscheduled times, and noted some bugs with the chat feature. Several participants who used the test kit reported being fearful of collecting the blood sample, and one participant had trouble with sending the kit back as they had lost the instruction sheet. Several participants suggested having video tutorials to help with the specimen collection and shipping process. One Chicago participant suggested adding resources for mental health, healthy relationships, and connections to the community. When asked whether they would use the app in the future, one participant in Chicago said the following:

Yes, 100%... It’s really—it’s good. I would–like yeah, I would advocate for it.

## Discussion

### Principal Findings

Sexual minority youth face numerous barriers to accessing HIV and STI prevention services, including stigma and lack of access to prevention clinics, and have expressed high levels of interest in mobile health (mHealth) interventions to support HIV and STI prevention, which may help address some of these barriers [31,32]. While a growing number of mobile apps are now being designed and tested to support HIV and STI testing, linkage to care, and PrEP uptake [33], few have been tailored specifically for youth. This study describes findings from 2 phases of formative research to develop and tailor the LYNX app to increase HIV and STI testing and PrEP uptake among YSMM in the United States and demonstrated high initial acceptability and feasibility of the app.

To increase user engagement with mobile apps, it has been suggested that developers use an iterative data collection process to obtain input from the target audience about app preferences [34]. Similar to other studies that used focus groups or theater testing as part of formative development [35-38], we conducted iterative focus groups with a cohort of YSMM to efficiently elicit feedback and inform the development of updated prototypes of the LYNX app. Overall, the app’s design was well-received by YSMM, and we received feedback to improve key components of the LYNX app, including the SexPro score and sexual diary, as well as recommendations for additional features to facilitate timely communication between users and staff and increase overall engagement and use of LYNX. In a 2-month technical pilot of the app, the revised LYNX app demonstrated feasibility based on use of the app as assessed via paradata and good acceptability as assessed via self-report.

Sanabria et al [39] used a multimethod, user-centered design approach, including input from a youth advisory board, a think-aloud protocol, and use case scenarios, to evaluate the usability of a mobile app to increase HIV testing among sexual and gender minority youth. This app, featuring HIV prevention information and an imaging algorithm for interpreting home-based HIV tests, demonstrated high user satisfaction and perceived usability that was independent of education and health literacy levels [40,41].

Several HIV risk scores have been developed and tested to assist in identifying individuals who may benefit from PrEP [42-45]; however, these have primarily been designed to be administered by providers and can be problematic, because clients may not be comfortable disclosing behaviors that put them at increased risk for HIV [46], and because identifying people at risk can be stigmatizing [47]. In this study, we tailored a patient-facing sexual health score (using a positive frame, with a higher score indicating higher sexual protection) specifically for use in youth. YSMM in our focus groups liked having both a color and numerical representation of protection from HIV; however, they wanted additional information on how the SexPro score was calculated and how they could improve their score. On the basis of these findings, key adaptations included adding information on the factors contributing to their score and how they could increase their score by changing behaviors or initiating PrEP.

Prior HIV research studies have also used sex diaries to capture detailed event-level sexual encounter data and to understand different profiles of HIV risk [48,49]; however, few have incorporated diaries into mobile apps as part of an mHealth intervention. A mobile app designed to collect sexual behavior in the Amsterdam PrEP Project was frequently used by SMM, with sexual behavior data collected by the app consistent with questionnaire data among those who used the app consistently [50]. In addition, in a study by Glick et al [51], completing sexual diaries was associated with reduced frequencies of reported anal sex, acquisition of new partners, and lower incident HIV and STI diagnoses. In our study, the sexual diary was included as part of the LYNX app as a strategy to help YSMM better understand and track their sexual behaviors, as well as increase engagement with the use of the app. In focus groups and the technical pilot, participants liked the diary feature; however, they made recommendations to tailor the language used in the sexual diary for YSMM and recommended an open-text section so the youth could customize their diary entries. The diary also appeared to be more useful for YSMM with a greater number of casual sexual partners. It will be important in future studies to characterize the types of partners YSMM enter into the app, for whom the diary is most useful, and whether diary use is sustained over time.

A key finding in our study was the importance of incorporating gamification features to increase engagement with the LYNX app by the youth. In a recent systematic review on the impact of mHealth-based gamification on the HIV prevention and care continuum in SMM by Luo et al [52], gamification interventions were shown to reduce condomless anal sex acts and were associated with an increase in PrEP adherence. Across the 26 studies included in the review, the most frequently used game components were points, followed by challenge, discussion forums, and mentorship or character narrative. In the LYNX app, we incorporated several gamification features based on feedback from YSMM in focus groups, including the ability to earn different badges, a “top 5 feature” for the most highly rated partners and sexual encounters, and a trends and insights page to synthesize the partner data they entered into the app into useful summaries. In other studies, the youth have also suggested that tangible versus virtual rewards (eg, a gift card) may be more likely to increase engagement [53], which should be explored in future studies.

Similar to other studies, most LYNX participants expressed interest in home HIV and STI self-collection kits, which could help overcome barriers to HIV testing among YSMM [31]. Convenience, privacy, and rapid result delivery have been cited by YSMM as principal motivators for using HIV self-tests [54]. In our study, YSMM expressed some concerns with collecting and preparing the specimens for shipment. The youth may benefit from videos demonstrating the specimen collection process and check-ins from staff on whether they need assistance with completing the test kits. On the basis of feedback in focus groups, we added a chat feature to facilitate communication between YSMM and staff and a button to call a clinician (eg, in the event of a positive HIV test), app features which have also been recommended in other studies of youth [55,56].

### Limitations and Strengths

This study had several limitations. First, the technical pilot was small and not designed to evaluate the efficacy of the intervention. In addition, due to the short duration of the pilot, we were unable to evaluate long-term feasibility and acceptability. Nonetheless, formative work through focus groups and optimizing usability in a technical pilot are critical steps in developing and tailoring interventions before testing efficacy in large randomized controlled trials. Social desirability may have impacted YSMM responses in the focus groups and technical pilot; however, we used a self-administered web-based survey to assess usability and acceptability and paradata metrics from the LYNX app to objectively assess feasibility. In addition, participants in focus groups and exit interviews were reminded that there were no right or wrong answers and were encouraged to provide honest feedback that would help us best improve the app. For both phases of this study, participants were enrolled in Chicago and Tampa, potentially limiting the generalizability of the findings, although both regions are in high-priority “ending the HIV epidemic” jurisdictions, and 1 site is located in the South, the region accounting for over half of new HIV diagnoses nationally. Despite these limitations, our study had several strengths, including enrolling a diverse cohort of YSMM across both sites; the user-centered approach, incorporating input from our participants into the final design of our intervention; and the mixed methods approach to evaluate feasibility and acceptability in our technical pilot.

### Conclusions

Using a user-centered design approach via iterative focus groups, we tailored the LYNX app to support HIV and STI testing and PrEP uptake among YSMM in the United States. Preliminary testing in a technical pilot demonstrated high acceptability and feasibility of the refined version of the LYNX app and supports further evaluation in an upcoming effectiveness trial.

## Abbreviations

ATN: Adolescent Medicine Trials Network for HIV Interventions
HIPAA: Health Insurance Portability and Accountability Act
IMB: Information-Motivation-Behavioral Skills
IRB: Institutional Review Board
iTech: innovative technology
mHealth: mobile health
PrEP: pre-exposure prophylaxis
SMM: sexual minority men
STI: sexually transmitted infection
SUS: System Usability Scale
YSMM: young sexual minority men
